# Dose-Related Pharmacokinetic and Pharmacodynamic Effects of Intramuscular Epinephrine in Healthy Neonatal Piglets

**DOI:** 10.3390/children12091180

**Published:** 2025-09-04

**Authors:** Marwa Ramsie, Po-Yin Cheung, Raza Hyderi, Shrieya Praveen, Tze-Fun Lee, Megan O’Reilly, Georg M. Schmölzer

**Affiliations:** 1 Centre for the Studies of Asphyxia and Resuscitation, Neonatal Research Unit, Royal Alexandra Hospital, Edmonton, AB T5H 3V9, Canadashrieya@ualberta.ca (S.P.);; 2 Department of Pediatrics, University of Alberta, Edmonton, AB T6G 2R3, Canada

**Keywords:** newborn, epinephrine, intravenous, intramuscular, pharmacokinetic, pharmacodynamic

## Abstract

**Highlights:**

**What are the main findings?**

- Intramuscular epinephrine produced dose-dependent cardiovascular effects in newborn piglets.
- A dose of 0.1 mg/kg IM epinephrine achieved hemodynamic and pharmacokinetic effects comparable to standard 0.02 mg/kg IV epinephrine, while the lower IM dose (0.02 mg/kg) was largely ineffective.

**What is the implication of the main finding?**

- IM epinephrine at an adequate dose may represent a feasible alternative to IV administration during neonatal resuscitation when vascular access is delayed.
- Adoption of IM epinephrine could simplify vasopressor delivery, requiring less training, equipment, and time in emergency settings.

**Abstract:**

Background: Epinephrine is currently the only vasopressor recommended during neonatal cardiopulmonary resuscitation (CPR). Rapid vasopressor administration is critical during CPR; however, establishing vascular access can take several minutes and requires extensive skills and/or training. The intramuscular (IM) route provides rapid drug administration and does not require special skills, training, or equipment. We aimed to compare various doses of IM epinephrine to intravascular (IV) epinephrine in a healthy neonatal piglet model. Method: Fifteen newborn piglets (1–3 days of age) underwent anesthesia, intubation via a tracheostomy, and randomization to 0.02 mg/kg IM epinephrine, 0.1 mg/kg IM epinephrine, or 0.02 mg/kg IV epinephrine. Hemodynamic and cardiac function parameters were continuously recorded throughout the experiment. Blood was collected prior to drug administration and throughout the experiment for pharmacokinetic and pharmacodynamic analysis. Results: Dose-dependent changes in hemodynamic and cardiac function parameters were observed following IM epinephrine administration. Greater changes were observed with 0.1 mg/kg IM epinephrine, while there were little to no changes with 0.02 mg/kg IM epinephrine. Pharmacokinetic parameters were not different between 0.02 mg/kg IV epinephrine or 0.1 mg/kg IM epinephrine. Conclusions: IM epinephrine dose of 0.1 mg/kg was more effective in producing systemic hemodynamic and cardiac function changes compared to the lower IM dose 0.02 mg/kg.

## 1. Introduction

Epinephrine, the only vasopressor recommended during neonatal resuscitation, is an endogenous catecholamine with chronotropic (increases heart rate), inotropic (increases cardiac contractility), lusitropic (myocardial relaxation), and vasoconstrictor properties [1,2]. However, the optimal route, timing, and dose remains unknown [3,4]. Current neonatal resuscitation guidelines recommend preferable intravenous (IV) or intraosseous (IO) administration of epinephrine, or endotracheal tube (ETT) administration while IV/IO access is being established [3]. However, establishing IV/IO/ETT access might take several minutes, thereby potentially reducing the odds of survival. Most importantly, early epinephrine administration is key; during pediatric out-of-hospital non-shockable cardiac arrest the odds of survival (Odds ratio (OR) 0.91; 95% confidence interval (CI) 0.81–1.01) decreases by 9% for every minute delay in epinephrine administration [5].

The intramuscular (IM) route of administration provides rapid drug administration and is universally recommended as the first-line treatment with epinephrine for anaphylaxis [6]. In a retrospective case analysis of anaphylactic adults, median (interquartile range—IQR) time from patient contact to epinephrine administration was significantly shorter with the IM route compared to IV (4 (3–5) minutes vs. 7 (5–9) minutes, respectively, *p* < 0.001) [7]. However, time to IV access is much longer in neonates. A retrospective case analysis of newborns without a detectable heart rate at birth reported a median time to IV insertion and epinephrine administration of 12 min (no standard deviation reported) [8]. Retrospective case analyses by Boddu et al. and Heathcote et al. reported similar median (IQR) time to IV access of 14 (9–20) minutes and 9 (7–14) minutes, respectively, in neonates requiring resuscitation [9,10].

Ease of IM injection and shorter time to administration makes it a potential alternative route of administration during neonatal resuscitation. In a pediatric piglet model (~2–5 weeks of age) of cardiac arrest, piglets were randomized to 0.1 mg/kg IM or 0.01 mg/kg IV epinephrine and rates of survival were similar in both groups [11]. However, in a case series of four neonatal lambs injected with 0.1 mg/kg IM epinephrine (via the deltoid) during CPR, plasma epinephrine concentrations failed to increase until after successful resuscitation [12]. Data on the effects of IM epinephrine on pharmacokinetics and pharmacodynamics in a neonatal model are lacking. Therefore, we aimed to determine the hemodynamic, cardiac function, pharmacokinetic and pharmacodynamic effects of IM epinephrine as well as elucidate its optimal dose in a healthy, non-cardiac arrest neonatal piglet model.

## 2. Methods

**Figure 1:**
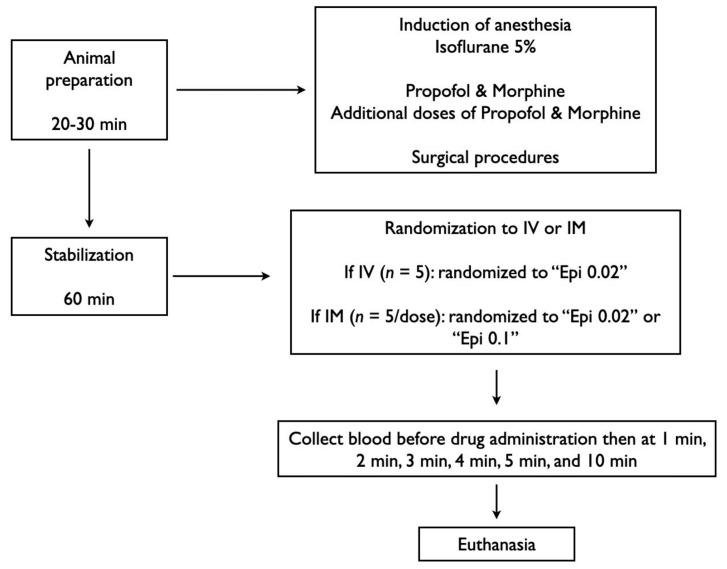
Study flow chart. Epi, epinephrine; IM, intramuscular; IV, intravenous.

**Figure 2:**
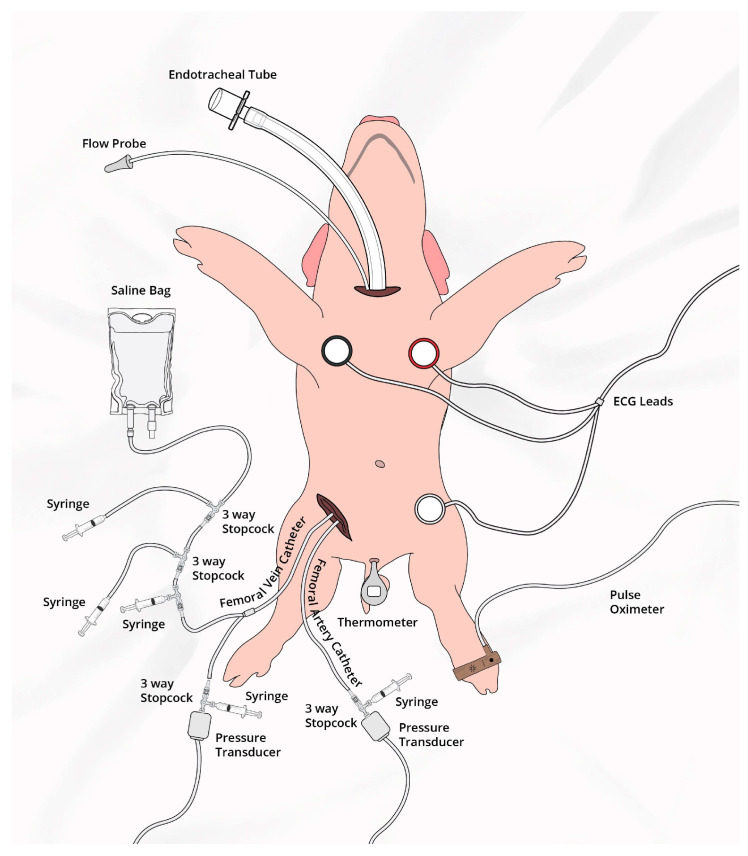
Schematic of animal experiment.

Fifteen newborn mixed-breed piglets (1–3 days of age, weighing 1.7–2.4 kg) were obtained on the day of experimentation from the University Swine Research Technology Centre. All experiments were conducted in accordance with the guidelines and approval of the Animal Care and Use Committee (Health Sciences), University of Alberta [AUP00002920], presented according to the ARRIVE 2.0 guidelines [13], and registered at preclinicaltrials.eu (PCTE0000630). A graphical display of the study protocol is presented in Figure 1 and a schematic display of the piglets instrumentation in Figure 2.

### 2.1. Inclusion and Exclusion Criteria

Newborn mixed-breed piglets 0–3 days of age with minimum weight of 1.5 kg were eligible. There were no exclusion criteria.

### 2.2. Randomization

Piglets were randomly allocated to IV Epinephrine (0.02 mg/kg) or IM Epinephrine (0.02 mg/kg or 0.1 mg/kg). IM doses were extrapolated from IV and ETT epinephrine dosing recommendations [3]. Allocation was block randomized with variable-sized blocks using a computer-generated randomization program (http://www.randomizer.org). Sequentially numbered, sealed, brown envelopes containing the allocation were opened during the experiment (Figure 1).

### 2.3. Blinding

TFL opened the randomization envelope and was solely responsible for epinephrine preparation and administration, remaining team members were blinded to group allocation. The statistical analysis was blinded to group allocation and only unblinded once completed.

### 2.4. Animal Preparation

Piglets were instrumented as previously described with modifications [14,15,16]. Following induction of anesthesia using isoflurane, piglets underwent a tracheostomy for intubation. Pressure-controlled ventilation (Sechrist Infant Ventilator Model IV-100; Sechrist Industries, Anaheim, CA, USA) was commenced at a respiratory rate of 16–20 breaths/min and pressure of 20/5 cmH_2_O. Oxygen saturation was maintained within 90–100%, while glucose was provided via an IV infusion of 5% dextrose at 10 mL/kg/h. IV propofol (5–10 mg/kg/h) and morphine (0.1 mg/kg/h) were provided to maintain anaesthesia, and additional IV doses of propofol (1–2 mg/kg) and morphine (0.05–0.1 mg/kg) were administered as needed. Piglet’s normothermic body temperature of 38.5–39.5 °C was maintained with a heating pad and overhead warmer.

### 2.5. Hemodynamic Parameters

5-French Argyle^®^ (Klein-Baker Medical Inc. San Antonio, TX, USA) single-lumen and double-lumen catheters were inserted via the right femoral artery and vein, respectively. The femoral arterial catheter was used for continuous arterial blood pressure monitoring in addition to arterial blood gas measurements and the venous catheter for administration of fluids and medications. The right common carotid artery was encircled with a real-time ultrasonic flow probe (2 mm; Transonic Systems Inc., Ithica, NY, USA) for measurements of cerebral blood flow. Cardiac function was continuously measured with a Millar^®^ catheter (MPVS Ultra1, ADInstruments, Houston, TX, USA) inserted into the left ventricle via the left common carotid artery [17,18].

Following surgical instrumentation, piglets were placed in the supine position and underwent a stabilization period (minimum one hour) to recover from surgical instrumentation until baseline hemodynamic measures stabilized. Ventilator rate was adjusted to maintain the partial arterial CO_2_ between 35 and 45 mmHg, as determined by periodic arterial blood gas analysis.

### 2.6. Cerebral Perfusion

The Invos^TM^ Cerebral/Somatic Oximeter Monitor (Invos 5100, Somanetics Corp., Troy, MI, USA) continuously measured cerebral oxygenation (crSO_2_), which calculates crSO_2_ and expresses values as the percentage of oxygenated hemoglobin (oxygenated hemoglobin/total hemoglobin). The sensors were placed on the right forehead of the piglet and secured with wrap and tape. A slim cap provided light shielding. Regional oxygen saturation values were recorded every second at a sample rate of 0.13 Hz [16].

### 2.7. Experimental Protocol

Following surgical instrumentation and stabilization, a subsequently numbered, sealed brown envelope containing the group allocation was opened (Figure 1). Piglets were randomized into three groups: 0.02 mg/kg IM Epi, 0.1 mg/kg IM Epi, or 0.02 mg/kg IV Epi. Piglets that were randomized to “IM” were administered one dose intramuscularly in the left inner thigh muscle. Arterial blood was collected before drug administration (baseline = time point “0 min”), 1, 2, 3, 4, 5, and 10 min after drug administration. Following final collection of blood, piglets were euthanized with an intravenous overdose of sodium pentobarbital (120 mg/kg).

### 2.8. Data Collection and Analysis

Demographics of study piglets were recorded. Heart rate, transonic flow probes, pressure transducer outputs, and Millar catheter were digitized and recorded with LabChart^®^ programming software (LabChart 7, ADInstruments, Houston, TX, USA). Plasma epinephrine concentrations and pharmacokinetic parameters were quantified and analyzed as previously described [19].

Normally distributed data are presented as mean (standard deviation—SD) and skewed data are presented as median (interquartile range—IQR). Data was tested for normality (Shapiro–Wilk and Kolmogorov–Smirnov test) and compared using either Student’s *t*-test (data normally distributed), Rank Sum if data were skewed, one- and two-way ANOVA with Dunnett post-test, or Fisher’s exact test for categorical variables. Both Hedges’ g and Cliff’s delta (δ) were reported as standardized measures of effect size as appropriate for parametric and non-parametric data, respectively. *p*-values are 2-sided and *p* < 0.05 was considered statistically significant. Statistical analyses were performed with SigmaPlot (Systat Software Inc, San Jose, CA, USA) and R version 4.3.2.

## 3. Results

15 newborn mixed-breed piglets were obtained on the day of the experiment (1–3 days of age, weighing 2.0 kg (±0.2 kg)). There were no differences in the baseline parameters between the groups except for age and diastolic arterial pressure (Table 1).

### 3.1. Hemodynamic Changes

**Figure 3:**
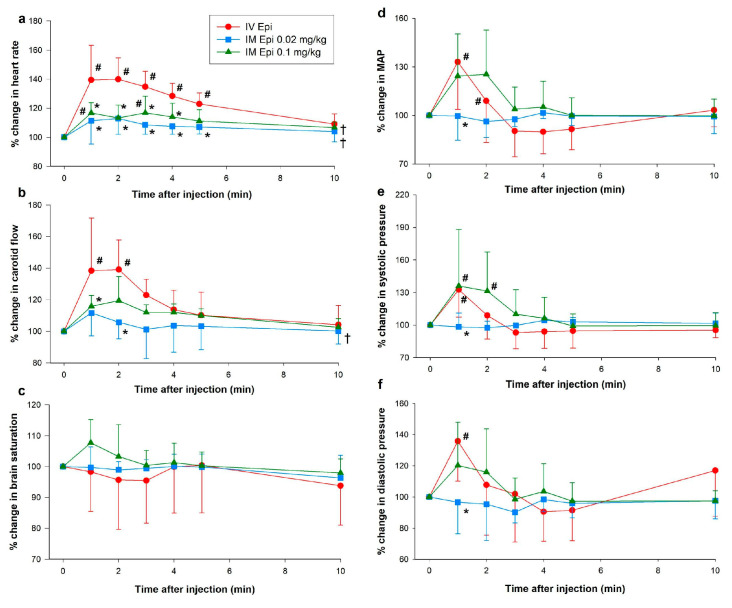
Percent change in heart rate, carotid blood flow, brain oxygen saturation, mean arterial pressure, systolic and diastolic pressure following intramuscular epinephrine administration. (**a**) percent change in heart rate; (**b**) percent change in carotid blood flow; (**c**) percent change in brain oxygen saturation; (**d**) percent change in mean arterial pressure (MAP); (**e**) percent change in systolic pressure; (**f**) percent change in diastolic pressure. Data are presented as mean % change from baseline (SD). # Significantly different from baseline; * Significantly different from IV epinephrine at the concurrent time point; † Significantly different from IV epinephrine over time (*p* < 0.05). Epi, epinephrine; IM, intramuscular; IV, intravenous.

**Figure 4:**
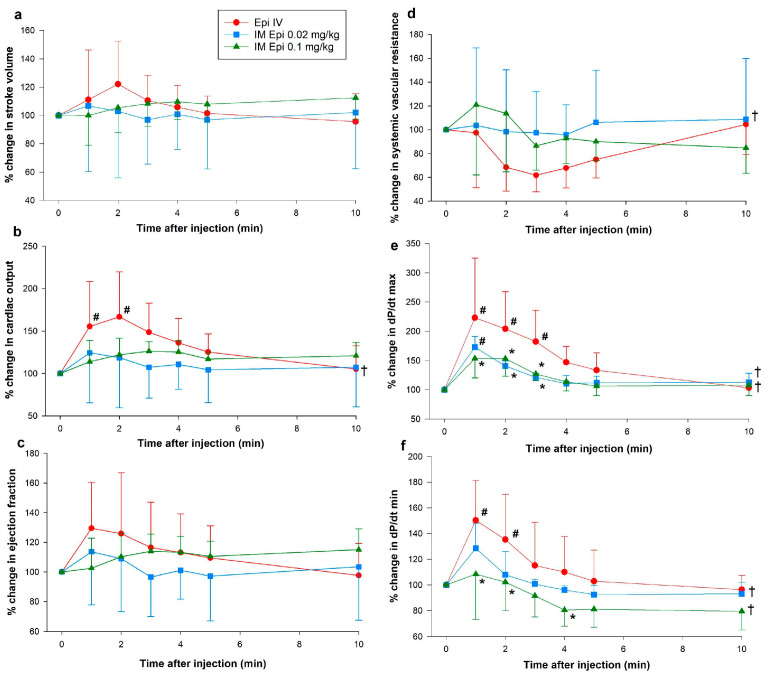
Percent change in stroke volume, cardiac output, ejection fraction, systemic vascular resistance, dp/dt_max_, and dp/dt_min_ following intramuscular epinephrine administration. (**a**) percent change in stroke volume; (**b**) percent change in cardiac output; (**c**) percent change in ejection fraction; (**d**) percent change in systemic vascular resistance; (**e**) percent change in dP/dt_max_; (**f**) percent change in dP/dt_min_. Data are presented as mean % change from baseline (SD). # Significantly different from baseline; * Significantly different from IV epinephrine at the concurrent time point; † Significantly different from IV epinephrine over time (*p* < 0.05). dp/dt_max_, maximum rate of left ventricular pressure change; dp/dt_min_, minimum rate of left ventricular pressure change; Epi, epinephrine; IM, intramuscular; IV, intravenous.

Except for increased dP/dt_max_ (maximum rate of left ventricular pressure change) one minute after administration, there were no significant changes from baseline in hemodynamic or cardiac function parameters with 0.02 mg/kg IM epinephrine. Both 0.1 mg/kg IM epinephrine and IV epinephrine had significant effects on hemodynamics and cardiac function (Figure 3 and Figure 4). While heart rate increased with 0.1 mg/kg IM epinephrine, values remained lower than with IV epinephrine (Figure 3a). Carotid blood flow was significantly lower with 0.1 mg/kg IM epinephrine one min after administration (118 (96–132)%) compared to IV epinephrine (134 (111–166)%, *p* = 0.036, Cliff’s δ = −0.36, 95% CI [−0.82, 0.38]), but had otherwise comparable flow throughout the experimentation period (*p* = 0.070). Changes in brain saturation, mean arterial pressure, and diastolic pressure were similar between 0.1 mg/kg IM epinephrine and IV epinephrine (Figure 3c,d,f). Systolic pressure was higher with 0.1 mg/kg IM epinephrine compared to IV epinephrine two minutes after administration (128 (107–177)% vs. 113 (89–126)%, respectively, *p* = 0.049, Cliff’s δ = 0.40, 95% CI [−0.50, 0.88]) and throughout the experimental period (*p* = 0.033) (Figure 3).

There were no significant changes from baseline in stroke volume, ejection fraction, or SVR in any groups (Figure 4a,c,d). Cardiac output increased one- (171 (103–200)%, *p* = 0.009) and two-min (145 (137–207)%, *p* = 0.04) after IV epinephrine administration but values returned to baseline after three min (Figure 3b). Left ventricular contractile function increased following 0.02, as shown by improved dP/dt_max_ (Figure 4e). Despite observed trends of improved ventricular contractility with IM epinephrine, these differences failed to reach statistical significance. IV epinephrine had significantly increased dP/dt_max_ and dP/dt_min_ (minimum rate of left ventricular pressure change) (Figure 4e,f).

### 3.2. Changes in Plasma Epinephrine Concentrations

**Figure 5:**
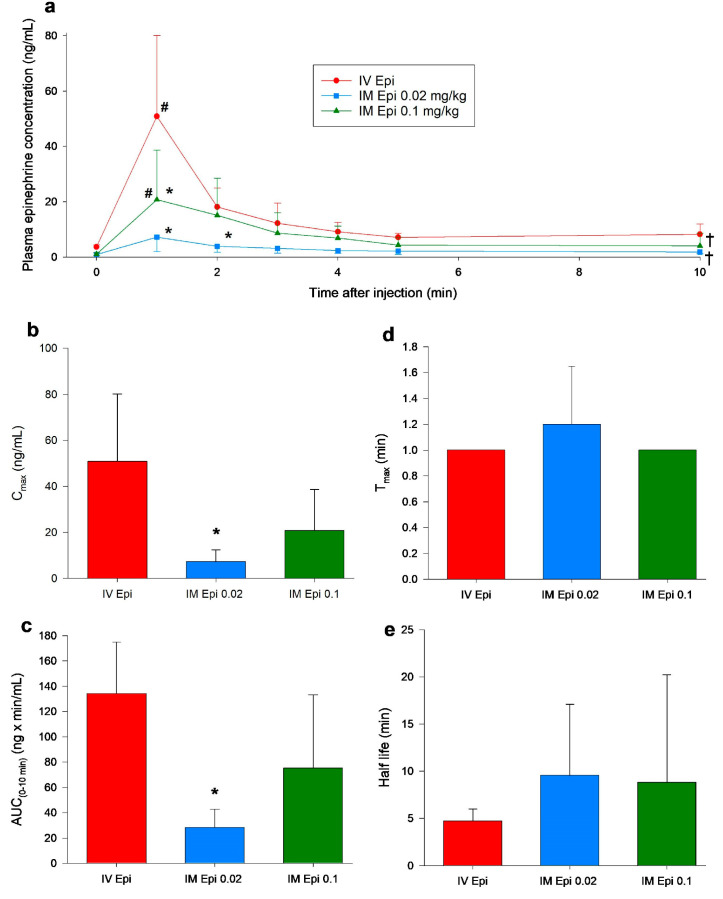
Plasma epinephrine concentrations and pharmacokinetics following intramuscular epinephrine administration. (**a**) plasma epinephrine concentration following intravenous and intramuscular administration; (**b**) *C_max_* (maximum plasma concentration) (ng/mL); (**c**) AUC (area under the curve) from 0 to 10 min (ng × min/mL); (**d**) *T_max_* (time to maximum plasma concentration) (minute); (**e**) half life (minute). Data are presented as mean (SD). # Significantly different from baseline; * Significantly different from IV epinephrine at the concurrent time point; † Significantly different from IV epinephrine over time (*p* < 0.05). Epi, epinephrine; IM, intramuscular; IV, intravenous.

Plasma epinephrine concentrations only slightly increased following 0.02 mg/kg IM epinephrine administration and were not significantly different from baseline values throughout the 10 min observation period (Figure 5a). Plasma concentrations were significantly increased from baseline one minute after administration of 0.1 mg/kg IM epinephrine (1.8 (0.8–8.3) ng/mL vs. 6.7 (3.4–16.9) ng/mL, respectively, *p* = 0.006, Cliff’s δ = 0.70, 95% CI [0.19, 0.91]) and IV epinephrine (3.6 (3.0–4.5) ng/mL vs. 43.6 (27.6–81.4) ng/mL, respectively, *p* < 0.001, Cliff’s δ = 1.00, 95% CI [0.97, 1.00]) (Figure 5a). Plasma epinephrine concentrations were significantly lower one minute after administration of 0.02 mg/kg IM (8.2 (3.3–17.1) ng/mL, *p* < 0.001, Cliff’s δ = −0.76, 95% CI [−0.97, 0.088]) and 0.1 mg/kg IM (16.9 (6.7–30.1) ng/mL, *p* < 0.001, Cliff’s δ = −0.52, 95% CI [−0.89, 0.26]) compared to IV epinephrine (43.5 (27.6–81.4) ng/mL) (Figure 5a). Two-minutes after administration of 0.02 mg/kg IM epinephrine, plasma concentrations remained lower then IV-treated piglets (4.1 (2.5–8.4) ng/mL vs. 16.8 (12.4–25.1) ng/mL, respectively, *p* = 0.027, Cliff’s δ = −0.92, 95% CI [−0.99, −0.52]) but were comparable for the remaining experimentation period (Figure 4a). Piglets administered 0.02 or 0.1 mg/kg IM epinephrine had lower plasma epinephrine concentrations over the experimental period compared to IV epinephrine (*p* < 0.001 and *p* = 0.004, respectively) (Figure 5a).

### 3.3. Pharmacokinetic Parameters

Maximum plasma concentration (*C_max_*), which occurred at one minute after administration, was significantly lower with 0.02 mg/kg IM epinephrine than the IV route (8.1 (2.6–11.5) ng/mL vs. 43.5 (27.6–81.4) ng/mL, respectively, *p* = 0.01, Hedges’ *g* = −2.06, 95% CI [−3.69, −0.43]) (Figure 4b). Time to maximum plasma concentrations (*T_max_*) and half-life were not different between 0.02 or 0.1 mg/kg IM epinephrine and IV epinephrine (Figure 5d,e). Area under the curve from baseline to 10 min (AUC_0–10_), a measure of the extent of drug absorption, was significantly lower with 0.02 mg/kg IM epinephrine (29 (17–39) ng·min/mL) compared to IV epinephrine (140 (92–170) ng·min/mL, *p* = 0.022, Cliff’s δ = −0.92, 95% CI [−0.99, −0.52]).

## 4. Discussion

Early epinephrine administration during cardiac arrest is associated with increased survival. In an analysis of pediatric patients with out-of-hospital non-shockable cardiac arrest, odds of survival was decreased by 57% (OR 0.43; 95% CI 0.16–1.14) when epinephrine was administered late (≥10 min) compared to early (<10 min) [5]. The IM route allows for rapid epinephrine administration and may serve as an alternative route of vasopressor administration during neonatal resuscitation. The current study is the first to systemically assess the dose-related pharmacokinetic and pharmacodynamic effects of IM epinephrine in a healthy neonatal piglet model. The results of our studies can be summarized as follows: administration of IM epinephrine produce dose-related changes in systemic and cardiac hemodynamics that are reflected in their pharmacokinetic profiles.

The 0.1 mg/kg dose of IM epinephrine had optimal hemodynamic effects and comparable pharmacokinetic parameters to IV epinephrine. There were little to no changes in hemodynamics or cardiac function following administration of 0.02 mg/kg IM epinephrine compared to IV epinephrine, likely due to low drug absorption as shown in Figure 5. Significant differences in pharmacokinetics between IV epinephrine and 0.02 mg/kg IM epinephrine, but not 0.1 mg/kg IM, suggests a higher dose is required for IM administration to achieve similar pharmacokinetics and pharmacodynamics as IV-administered epinephrine.

Berkelhamer et al. injected 0.1 mg/kg IM epinephrine into the deltoid muscle of four neonatal lambs during asphyxia-induced asystole and reported plasma epinephrine concentrations failed to increase until after successful resuscitation [12]. In contrast, Mauch et al. randomized pediatric piglets to 0.01 mg/kg IV or 0.1 mg/kg IM epinephrine (via the tongue) during cardiac arrest and reported similar rates of survival between the groups (100% vs. 87.5%, respectively, *p* > 0.05) [11]. Similarly, O’Reilly et al. randomized pediatric piglets to 0.3 mg IM epinephrine (via the left outer thigh) or 0.02 mg/kg IV epinephrine and reported similar rates of ROSC between bradycardic (3/5 (60%) vs. 3/5 (60%), respectively, *p* = 1.00) and asystolic (2/5 (40%) vs. 1/5 (20%), respectively, *p* = 1.00) piglets [20]. The differences in plasma epinephrine concentrations between our study, Berkelhamer et al. and Mauch et al. may be due to differences in injection location, in addition to the species and developmental differences in metabolism. In the current study we injected IM into the left inner thigh while Berkelhamer et al. injected into the deltoid muscle and Mauch et al. injected into the tongue (well-perfused muscle). Bioavailability of IM epinephrine is dependent on the muscle being injected, with one study reporting five-times higher peak epinephrine concentrations when injected into the vastus lateralis compared to the deltoid muscle [21].

## 5. Limitations

Piglets have several anatomical and physiological similarities to humans that make them an ideal neonatal model [22,23], as such we expect the IM injection site and doses used in this study to produce similar effects in human neonates; however, several limitations should be considered. Our model uses piglets that have undergone the fetus to neonate transition and do not possess fetal features (i.e., no lung fluid present or a patent ductus arteriosus). Additionally, our model uses piglets that were sedated/anesthetized, and intubated with a tightly sealed endotracheal tube to prevent leak, which may not occur in the delivery room. As our study only examined changes that occur immediately following drug administration, potential changes that may occur hours afterwards could not be examined. Although team members were blinded to group allocation, we were unable to blind the intervention due to the difference in administering IM versus IV epinephrine. Furthermore, our study examined IM epinephrine in healthy post-transitional piglets with intact circulation and will need to be replicated in a cardiac arrest model. While significant changes in hemodynamics and pharmacokinetics were observed with 0.1 mg/kg IM epinephrine in the current study, during scenarios of poor muscle perfusion (such as cardiac arrest) IM epinephrine bioavailability may be reduced. Investigation on the optimal IM epinephrine dose during cardiac arrest is warranted and a dose greater than 0.1 mg/kg may be warranted. An ongoing randomized controlled trial evaluating early IM epinephrine in pediatric out-of-hospital cardiac arrest is using a dose of 0.3 mg IM epinephrine for 3–5 kg infants [24].

## 6. Conclusions

In this dose–response study in healthy neonatal piglets, 0.1 mg/kg IM epinephrine had comparable pharmacokinetics and pharmacodynamics compared to 0.02 mg/kg IV epinephrine. The lower IM epinephrine dose of 0.02 mg/kg had significantly lower pharmacokinetic parameters compared to the same IV dose.

## Figures and Tables

**Table 1:** Baseline characteristics.

	IV Epi 0.02 mg/kg (n = 5)	IM Epi 0.02 mg/kg (n = 5)	IM Epi 0.1 mg/kg (n = 5)	*p*-Value
Age (days)	2 (2–2)	2 (2–2)	3 (3–3)	0.024
Weight (kg)	2 (1.9–2.2)	1.9 (1.8–2)	2 (1.8–2.2)	0.423
Sex (male/female)	5/0	3/2	3/2	0.451
pH	7.45 (7.44–7.51)	7.48 (7.48–7.49)	7.48 (7.46–7.48)	0.750
PaCO_2_ (torr)	35 (30–35)	31 (31–33)	30 (29–32)	0.327
PaO_2_ (torr)	80 (71–81)	69 (65–74)	73 (72–74)	0.867
Base excess (mmol/L)	0.8 (0.7~2)	−0.3 (−0.3~0.6)	−1.7 (−1.8~−0.5)	0.119
Lactate (mmol/L)	4.73 (4.14–6.49)	4.83 (4.63–5.37)	6.27 (5.82–6.29)	0.694
Na^+^ (mmol/L)	136 (135–137)	138 (136–140)	126 (130–137)	0.134
Hemoglobin (g/dL)	8.3 (8.1–8.5)	7.9 (7.5–8.1)	7 (5.9–8.9)	0.370
Heart rate (bpm)	186 (171–199)	180 (163–192)	205 (203–237)	0.276
Mean arterial pressure (mmHg)	61 (61–67)	64 (63–65)	71 (70–74)	0.088
Systolic (mmHg)	86 (85–89)	89 (85–91)	95 (87–96)	0.943
Diastolic (mmHg)	45 (42–52)	45 (44–48)	60 (53–67)	0.009
Carotid blood flow (mL/kg/min)	90 (72–93)	81 (65–90)	92 (74–104)	0.530
Cerebral oxygenation (%)	48 (48–50)	42 (39–46)	41 (40–47)	0.145
Stroke volume (mL/kg/min)	1.73 (1.54–2.97)	1.44 (1.31–2.21)	1.90 (1.42–2.41)	0.512
Cardiac output (mL/kg/min)	322 (256–640)	294 (220–429)	338 (312–558)	0.584
Ejection fraction (%)	53 (44–75)	33 (23–49)	49 (40–58)	0.105
Systemic vascular resistance	0.21 (0.10–0.22)	0.18 (0.13–0.28)	0.19 (0.14–0.21)	0.841
dP/dt max (mmHg)	2680 (2540–4392)	2868 (2454–3230)	4224 (3741–5403)	0.033
dP/dt min (mmHg)	−3959 (−3989~−2946)	−3030 (−3749~−2215)	−4799 (−6240~−3513)	0.054

Data are presented as median (IQR); Na^+^—sodium, PaCO_2_—partial pressure of arterial carbon dioxide, PaO_2_—partial pressure of arterial oxygen.

## Data Availability

The datasets generated and analyzed for this study are available from the corresponding author (GMS), upon reasonable request.
